# *Arteria lusoria* dyspnéisante: à propos d’un cas

**DOI:** 10.11604/pamj.2020.37.318.23253

**Published:** 2020-12-07

**Authors:** Kader Ndiaye, Adamou Abbassi, Sory Traoré, Jacob Vagba, Aboubacar Aouami, Martine Berret

**Affiliations:** 1 Service d´Anesthésie et Réanimation, Centre Hospitalo-Universitaire la Renaissance, N´Djamena, Tchad,; 2 Service de Radiologie et Imagerie Médicale, Centre Hospitalo-Universitaire la Renaissance, N´Djamena, Tchad

**Keywords:** *Arteria lusoria*, dyspnée laryngée, rapport de cas, Arteria lusoria, laryngeal dyspnea, case report

## Abstract

L´arteria lusoria ou artère sous Clavière droite retro-œsophagienne constitue la malformation de l'arc aortique la plus fréquente avec une prévalence de 0,5-2,5 %. Elle peut être découverte face à des symptômes de compression des voies respiratoires et/ou de l’œsophage tels qu´une dyspnée ou une dysphagie voire infections respiratoires à répétition; mais la plupart des cas c´est une pathologie asymptomatique comme rapportée par plusieurs auteurs. Nous rapportons le cas d´une patiente de 44 ans qui a été prise en charge dans notre service de réanimation pour une détresse respiratoire liée à l´arteria lusoria. C´est une cause rare de dyspnée à laquelle il faudrait y penser devant une dyspnée ne répondant pas au traitement médical. Sa prise en charge reste médicale avec une simple surveillance, si elle est asymptomatique mais une intervention chirurgicale serait nécessaire si elle devient symptomatique ou quand elle est associée à un diverticule de Kommerell (DK).

## Abstract (en)

Arteria lusoria or retroesophageal right subclavian artery is the most common aortic arch malformation, accounting for 0.5-2.5% of cases. It can be detected in patients with symptoms including airway and/or esophageal compression such as dyspnea or dysphagia or even recurrent respiratory infections; but it is mostly asymptomatic, as reported by several authors. We here report the case of a 44-year-old female patient treated in our emergency room due to respiratory distress syndrome associated with arteria lusoria. This is a rare cause of dyspnea which should be suspected in patients with dyspnea not responding to medical treatment. Asymptomatic patients undergo medical treatment associated with simple surveillance. Surgery is necessary when it becomes symptomatic or when it is associated with Kommerell diverticulum (KD).

## Introduction

L´*arteria lusoria* ou artère sous clavière droite retro-œsophagienne constitue la malformation de l'arc aortique la plus fréquente, elle peut être associée à d'autres anomalies congénitales du cœur et des gros vaisseaux, notamment le tronc bi carotidien qui constitue un tronc commun donnant naissance aux deux artères carotides primitives [1]. L´arc aortique gauche avec artère sous-clavière droite aberrante ou *arteria lusoria*, est l´anomalie de l´arc aortique la plus fréquente, avec une prévalence de 0,5-2,5% [2]. Les anomalies de l´arc aortique, relativement fréquentes, représentent 15 à 20% de toutes les maladies cardiovasculaires congénitales [3]. Elles peuvent être découvertes lors des symptômes de compression des voies respiratoires et/ou de l’œsophage [3]; la plupart des cas sont asymptomatiques de découverte fortuite [1]. Nous rapportons un cas d´*arteria lusoria* dyspnéisante découverte au scanner chez une patiente de 44 ans.

## Patient et Observation

**Figure 1:**
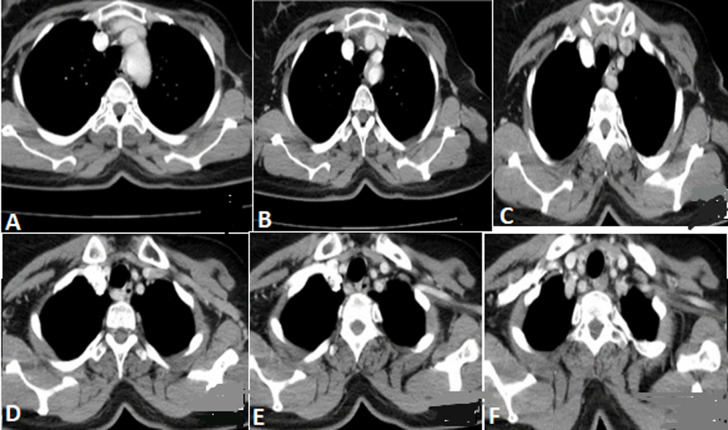
*arteria lusoria;* progression (A, B, C, D, E, F) par ordre depuis l´émergence aortique gauche jusqu´à la droite

Il s´agit d´une patiente âgée de 44 ans qui a été admise dans notre service de réanimation pour la prise en charge d´une dyspnée laryngée. L´épisode actuel serait survenu trois jours avant son hospitalisation, marqué par la survenue d´une dyspnée de type laryngé d´aggravation progressive. Elle nous a été référée pour une meilleure prise ne charge de cette dyspnée d´aggravation croissante. Dans ses antécédents on retrouve une notion des épisodes récurrents de dyspnée et deux chirurgies cervicales respectivement thyroïdienne et masse latéro-cervicale; elle était sous traitement hormonal thyroïdien substitutif. A l´examen clinique, on note une détresse respiratoire de type laryngée marquée par un tirage intercostal, un balancement thoraco-abdominal. Les examens cardio-vasculaire et neurologique étaient sans particularité. Un scanner cervico-thoracique objective une artère sous clavière droite lusoria passant derrière l´œsophage avec une potentielle compression de l´œsophage ainsi que de la trachée (Figure 1); par ailleurs il ne montre pas d´anomalie des parties molles cervicales ni processus tumoral cervical et il n´y´ a pas d´anomalie biologie hormis une THS us élevée. Le traitement a consisté en une prise en charge urgente en réanimation nécessitant une intubation orotrachéale avec une ventilation contrôlée sous sédation, une réhydratation hydro-électrolytique, ainsi qu´une corticothérapie à forte dose à raison de 240 mg de methyl-prédnisolone en IV (soit 3 mg/kg par jour). L´évolution a été marquée par une nette régression de la dyspnée, ce qui a permis son extubation à J2 avec une voix qui est restée rauque et persistance d´une faible dyspnée laryngée puis une nette amélioration sous nébulisation de corticoïde (pulmicort*). Nous l´avons référée vers un centre disposant un plateau technique pour une prise en charge complémentaire chirurgicale.

## Discussion

Les arcs pharyngés et aortiques sont développés à partir de la 4^e^semaine d´aménorrhée (SA). Les arcs aortiques dérivés du sac aortique ventralement et des aortes dorsales paires dorsalement six paires d´arcs aortiques se développent et ne sont pas tous présents en même temps. Il y´aurait une involution des deux premiers arcs aortiques. Ainsi le 3^e^arc donne naissance aux artères carotides primitives; le 4^e^arc donne l´artère sous clavière droite, la crosse aortique et l´artère sous clavière gauche. Le 6^e^arc donne les artères pulmonaires. La configuration normale rencontrée dans 70% des cas [4]. L´arc aortique gauche avec artère sous-clavière droite aberrante ou *arteria lusoria*, est l´anomalie de l´arc aortique la plus fréquente avec une prévalence de 0,5-2,5%. Quatre vaisseaux proviennent séquentiellement de l´arc aortique: l´artère carotide commune droite, l´artère carotide commune gauche, l´artère sous-clavière gauche et l´artère sous-clavière droite aberrante, qui se croisent vers le haut et la droite dans le médiastin postérieur. Elle résulte d´une disparition dans le remodelage complexe des arcs branchiaux, typiquement de l´aorte dorsale droite distalement à la sixième artère inter segmentaire cervicale [2]. Carles D *et al*. ont montré que 2% des individus dans la population générale présentent une artère sous-clavière droite aberrante [5].

Les variations embryologiques des arcs aortiques sont les plus souvent asymptomatiques. Néanmoins, elles se compliquent de symptômes trachéo-œsophagiens. Dans la majorité des cas, c´est l´artère sous-clavière droite rétro-œsophagienne aberrante (*arteria lusoria*) qui est à l´origine d´une compression de la trachée pouvant se manifester par une dyspnée. Plus rarement, une artère sous-clavière gauche rétro-œsophagienne aura les mêmes conséquences et se manifestera par de la dysphagie. Les signes peuvent apparaître tardivement, parfois à l´âge adulte; au contraire, chez les nourrissons et jeunes enfants, les anneaux vasculaires se manifestent plutôt par des symptômes respiratoires [3]. Fanette J rapporte dans sa série portant sur l´étude morphodensitométrique de 150 cas et Applications cliniques, que l´*arteria lusoria* devient symptomatique chez les adultes, généralement au cours de la quatrième ou cinquième décennie de la vie, mais l´interrogatoire retrouve souvent des troubles respiratoires à répétition dans l´enfance, évoquant une compression trachéale ancienne et spontanément régressive [6]. Les observations de Solowianiuk M [3] et Fanette J [6] concordent avec l´âge de notre patiente qui est dans cette tranche d´âge.

La plupart des cas c´est une pathologie asymptomatique comme rapporté par plusieurs auteurs dont Benhaddah A *et al*. ont découvert fortuitement un cas d´*arteria lusoria* lors de la réalisation d´un scanner thoracique pour traumatisme thoracique chez une patiente de 50 ans; ce qui a mis en évidence une artère aberrante rétro-œsophagienne il s´agit d´une *arteria lusoria* droite [7]. Sur le plan clinique, l'*arteria lusoria* est souvent asymptomatique, parce que cette dernière ne forme pas d'anneau complet autour de la trachée, elle est découverte dans la majorité des cas de façon fortuite lors d'une exploration thoracique pour d'autres pathologies. L´*arteria lusoria* devient symptomatique dans trois cas: d'une part quand la trachée et l´œsophage sont comprimés entre l'*arteria lusoria* en arrière et le tronc bicarotidien en avant et d´autre part lorsqu'il existe un anévrysme de cette artère qui constitue une complication redoutable, et enfin avec l´âge lors d'une dégénérescence athéroscléreuse de l'artère, ou de la survenue d'une dysplasie fibro-musculaire décrite par Klinkhamer AC *et al*. [8] rapporté par Khnaba S *et al*. [1]. Quant à Stone MM. *et al*. dans leur série sur la prise en charge des artères sous-clavières aberrantes: (66%) anomalies étaient diagnostiquées de manière fortuite, mais huit (33%) avaient des symptômes [9].

Les maladies cardiovasculaires congénitales touchent environ 1% des enfants nés vivants [3]. Dans 15 à 20% des cas, il s´agit d´une anomalie de l´arc aortique. Comme les anomalies de l´arc aortique peuvent entraîner des symptômes respiratoires chroniques qui ne répondent pas au traitement médicamenteux, il est indispensable de les évoquer lors du diagnostic différentiel [3]. Michal P *et al*. dans leur étude sur 141 cas en étudiant les aspects morphologiques et cliniques de l'une des variations les plus importantes - une étude systématique de 141 rapports, ils ont trouvé une compression sous-jacente d´une *arteria lusoria* droite avec une dyspnée dans 18,7% c des cas [10]. Dans notre cas la patiente présentait des épisodes récurrents de dyspnée laryngée, malgré les deux interventions chirurgicales qu´elle avait eues pour le même motif. Le diagnostic n´a été mis en évidence que durant nos investigations explorationnelles, notamment lors de la réalisation d´un scanner cervico-thoracique qui a mis en évidence une *arteria lusoria* avec potentielle compression trachéale. Un traitement corticoïde primaire a permis d´améliorer l´état clinique initial. La prise en charge est chirurgicale si elle devient symptomatique soit en présence d´une dyspnée lusoria ou infections respiratoires chroniques, soit d´une dysphagie lusoria. Stone MM *et al*. ont constaté que dans 29% des cas un diverticule de Kommerell (DK) était l'anomalie la plus fréquemment associée et la présence d´un DK nécessite une intervention du fait de symptômes ou d'une dégénérescence anévrysmale; l´évolution globale était favorable [9].

## Conclusion

En réanimation, on doit penser à une dyspnée lusoria devant des symptômes respiratoires récurrents, surtout ne répondant pas au traitement médical. Sa prise en charge nécessite souvent une simple surveillance et elle devient chirurgicale en présence des symptômes ou l´association avec un diverticule de Kommerell. L´évolution est souvent favorable après une prise en charge précoce et adéquate.

## References

[1] Safae Khnaba, Meryem Menany, Mouna Moukinebillah, Touriya Amil, Bouchayb Radouane (2015). Arteria lusoria associée à un tronc bi carotidien: à propos d´un cas et revue de la littérature. Pan African Medical Journal.

[2] Myers PO, Fasel JHD, Kalangos A, Gailloud P (2010). Arteria lusoria: Developmental anatomy, clinical, radiological and surgical aspects. Ann Cardiol Angeiol.

[3] Solowianiuk M, Soulatges C, Farhat N, Holzki J, Seghaye MC (2016). Quand une anomalie encerclante des arcs aortiques se cache derrière des symptômes respiratoires et digestifs de l´enfant. Rev Med Liège.

[4] Myers PO (2009). L´arc Aortique: Embryologie, anatomie et variantes anatomiques pour le clinicien. Thèse de Doctorat en Médecine, Faculté de médecine, Université de Genève Suisse.

[5] Carles D, Pelluard F, André G, Nocart N, Sauvestre F (2014). L´artère sous clavière droite aberrante (arteria lusoria) et le risque de trisomie 21: analyse rétrospective de 11479 examens foetopathologiques. J Gynecol Obstet et Biol Reprod.

[6] Fanette Jeannon (2011). Arteria Lusoria: étude morphodensitométrique de 150 cas ; applications cliniques. Sciences du Vivant [q-bio].

[7] Benhaddad A, Bayoud A, Mahdadi S (2018). Arteria lusoria À propos d´un CAS. Morphologie.

[8] Klinkhamer AC (1966). Aberrant right subclavian artery: clinical and roentgenologic aspects. Am J Roentgenol Radium Ther Nucl Med.

[9] William MS, Joseph JR, Richard JF, Nitin G, Thomas CB, Samuel RM (2011). Prise en charge actuelle des artères sous-clavières droites aberrantes. Ann de Chir Vasc.

[10] Michal P, Lukasz C, Jaroslaw DK, Ludomir S, Miroslaw T, Agata M (2014). The Aberrant right subclavian artery (Arteria Lusoria): the morphological and clinical aspects of one of the most Important variations-a systematic study of 141 Reports. The scientific word journal. 2014;.

